# Retraining the veterans health administration’s REACH VET suicide risk prediction model for patients involved in the legal system

**DOI:** 10.1038/s44184-025-00143-9

**Published:** 2025-07-10

**Authors:** Esther L. Meerwijk, Andrea K. Finlay, Alex H. S. Harris

**Affiliations:** 1 VA Health Systems Research, Center for Innovation to Implementation (Ci2i), VA Palo Alto Health Care System, Menlo Park, CA USA; 2 VA National Center on Homelessness Among Veterans, Tampa, FLA USA; 3 Division of Health Systems Science, Department of Medicine, UMass Chan Medical School, Worcester, MA USA; 4 Stanford–Surgery Policy Improvement Research and Education Center (S-SPIRE), Department of Surgery, Stanford University School of Medicine, Palo Alto, CA USA

**Keywords:** Predictive medicine, Disease prevention, Preventive medicine

## Abstract

Although patients with criminal legal system involvement have among the highest rates of suicide, the model that identifies patients at high risk of suicide at the United States Veterans Health Administration (VHA) does not include predictors specific to criminal legal system involvement. We explored whether the model’s predictive ability would be improved (1) by retraining the model for legal-involved veterans and (2) by adding additional predictors associated with legal-involvement. For a combined outcome of suicide attempt or suicide death, the retrained models showed a positive predictive value (PPV) of 0.124 and false negative rate (FNR) of 0.527. Adding additional predictors associated with being legal-involved did not improve predictive accuracy. Retraining the VHA suicide risk prediction model for legal-involved patients improves the model’s predictive ability for this group of high-risk patients, more so than adding predictors associated with being legal-involved. A similar approach for other high-risk patients is worth exploring.

## Introduction

Among adults living in the United States (US), almost 19 million are military veterans^1^. Among them, almost 6 million (31.2%) veterans received services from the Veterans Health Administration (VHA). Veterans with involvement in the criminal legal system – hereafter referred to as ‘legal-involved veterans’ – are at increased risk of suicide and suicide attempts^2^. In 2021, the most recent year for which suicide rates are currently available, legal-involved veterans who had contact with VHA Veterans Justice Programs had among the highest annual suicide rates: about 150 per 100,000 compared to about 40 per 100,000 for other VHA patients^1^. For comparison, the annual suicide rate for non-veteran US adults was 18 per 100,000.

For years, suicide prevention among military veterans has been one of the highest priorities for VHA^3,4^. The multiple efforts to prevent suicide include the Recovery Engagement and Coordination for Health–Veterans Enhanced Treatment (REACH VET) program, which is a nationwide outreach program informed by a suicide risk prediction model that identifies VHA patients at high risk of suicide^5,6^. The prediction model is run monthly and predicts each patient’s risk of suicide during the next 30 days. Patients with a risk score in the top 0.1% - about 6800 patients per month in 2021 - are flagged as high risk and receive care under the REACH VET program. It is important to note that suicidal patients who are at lower risk, which may still be high just not high enough to make the top 0.1%, do receive care as described in VHA’s clinical practice guideline for assessment and management of patients at risk of suicide^7^. A quasi-experimental evaluation of the REACH VET program found its implementation to be associated with increases in completed outpatient appointments and proportion of individuals with new safety plans, as well as reductions in mental health admissions, emergency department visits, and suicide attempts. However, the program was not found to be effective in reducing death by suicide or all-cause mortality^8^.

The risk prediction model that underlies the REACH VET program is a logistic regression model using predictors based on prior suicidal behavior, medical diagnoses, prescription medication, and healthcare utilization during the two years that precede the model run date, or index date. These data are available from VHA electronic health records. The original REACH VET model included 350 individual predictors plus interaction terms^5^. The majority of those variables were dropped after an evaluation showed that very similar performance could be achieved with a more parsimonious model containing about 60 predictor variables^6^. Suicide is a complex phenomenon^9^ and suicide risk prediction models are notorious for generating many false positives (people who are flagged but will not die by suicide) and high rates of false negatives (people who are not flagged but who will die by suicide)^10,11^. The REACH VET suicide risk prediction model is no exception and has a reported sensitivity of 2.2% for patients classified as high risk (the top 0.1%)^6^, meaning that the vast majority of suicides occurred among patients who were considered at lower risk.

In analyses of the REACH VET model based on 2018 data^12^, it was found that legal-involved veterans represented 11.8% of the high-risk group, which is well above the estimated 0.8% of legal-involved veterans patients in the general VHA patient population. It is worth noting that the REACH VET model does not include a predictor for being legal involved^6^. Also, better predictive performance was achieved by predicting a combined outcome of death by suicide or suicide attempt, although this finding was observed with a model that was not specifically trained for that combined outcome. A narrative review of empirical evidence concluded that clinical factors predict suicide attempts and deaths only in so far as they predict suicide ideation^13^. While there are differences in risk factors associated with suicide attempts and suicide deaths, e.g., history of non-suicidal self-injury, an extensive meta-analysis also found overlap, most notably demographic factors, treatment history, and prior suicide attempts^14^. As a prior suicide attempt is *the* most important predictor of death by suicide^9,15^, exploring models that predict the combined outcome of death by suicide or suicide attempt does not seem unreasonable, especially because death by suicide on its own is a low base rate event that is extremely hard to predict^16^.

Given that legal-involved veteran patients are a higher proportion of high-risk patients identified by the REACH VET model and that the REACH VET model does not currently include predictors that are specific to legal-involved veteran patients, the goals of this study were 1) to explore whether retraining the REACH VET model for legal-involved veteran patients would improve its ability to identify patients who either died by or attempted suicide, and 2) to explore whether additional predictors associated with being legal-involved would improve predictive ability in this population.

## Methods

In this cohort study, we retrained the REACH VET model on multiple cohorts of patients alive at the start of each of five purposely selected months to capture variation in the outcome across 2021: March, May, July, September, and November. At the time of the study, 2021 was the most recent year for which outcome data were available. Models trained on all VHA patients hereafter are referred to as full models, whereas models trained on only legal-involved veteran patients are referred to as legal-involved veteran models. The study was approved by the Stanford University Institutional Review Board and the Research and Development Committee of the VA Palo Alto Health Care System.

### Patient cohort specifications

All cohorts used in this study consisted of VHA patients with inpatient or outpatient encounters during two years prior to the cohort index date, almost 7 million patients each month. Patients were coded as involved in the legal system if 1) their records in VHA’s Corporate Data Warehouse (CDW), which houses clinical and administrative records for all VHA patients, indicated at least one outpatient visit with the Veterans Justice Programs Health Care for Re-entry Veterans (VHA stop code 591) or Veterans Justice Outreach (VHA stop code 592) during the year before the cohort index date, or 2) if they received services for homelessness during the year before the cohort index date and were assessed for being involved in the legal system. The latter was based on records from the Homeless Operations Management and Evaluation System (HOMES), which were available in CDW. The Healthcare for Re-entry Veterans program primarily serves incarcerated veterans exiting prison and re-entering community living. The Veterans Justice Outreach programs primarily serve Veterans who have encounters with law enforcement in the community, have been arrested and are in jail custody, or are involved in court proceedings.

### Predictor variables

Data for predictor variables in the REACH VET model were determined with SQL scripts initially created to compute historical REACH VET scores and that were provided and validated by the VA Serious Mental Illness Treatment Resource and Evaluation Center. These scripts pulled predictor data from CDW. As the data for March 2021 included all 0 values for inpatient mental health days during 7 months before the index and any mental health discharges 1, 6, and 12 months before the index, these four predictor variables were dropped from the models. Also, a perfect correlation existed between the number of days any health services were used during 7 months before the index and the number of days outpatient health services were used during 7 months before the index. We opted to drop the number of days any health services were used during 7 months before the index from the models to avoid numeric instability. Supplementary information Table S1 shows the March 2021 retrained REACH VET model to illustrate the variables included in the models.

An initial analysis to address our second goal indicated that simply adding a flag for being legal-involved as predictor did not change predictive ability of the REACH VET model, one way or the other. Therefore, based on communication by one of our co-authors with a VA suicide prevention program manager following an internal and unpublished quality improvement program, we explored the following clinician-identified predictors associated with being legal-involved: presence of family discord, presence of high pain, a positive screen on the Columbia Suicide Severity Rating Scale^17^, and prior suicide attempts resulting in injury. With the exception of suicide attempts resulting in injury, these predictors were not included before in the REACH VET model^6^. Suicide attempts resulting in injury is a refinement of the broadly defined suicide attempt predictor that is part of the REACH VET model.

Family discord was based on ICD-10 diagnosis codes Z63.0, Z63.1, Z63.5, Z63.8, and Z63.9. Patients with either of these codes recorded in CDW during one month before the index date were considered positive for the presence of family discord.

High pain was based on scores recorded in CDW for pain assessed on a numerical rating scale ranging from 0 to 10. Patients with a score of 7 or higher anywhere during one year before the index date were considered positive for high pain. This cut-off corresponds with severe pain in patients with chronic musculoskeletal pain and low catastrophizing tendency^18^.

Screening results with the Columbia Suicide Severity Rating Scale are recorded in CDW as part of mental health assessments. A positive screen indicates an affirmative response on any of four questions assessing suicidal plans and intent.

Patients with a recorded suicide attempt that resulted in injury during one year before the index date were considered positive for this variable. The consolidated records from suicidal behavior and overdose reports and data from the Suicide Prevention Action Network^19^ available in CDW indicate whether a suicide attempt resulted in injury.

### Outcome variable

For all analyses in this study, we used the combined outcome of death by suicide or suicide attempt. To determine death by suicide, we used cause of death data from the VA-DoD Mortality Data Repository^20^. Suicide attempts were based on ICD-10 diagnosis codes as well as the consolidated records from suicidal behavior and overdose reports and data from the Suicide Prevention Action Network^19^, all of which were available in CDW. Deaths by suicide and suicide attempts were determined during 30 days following the index date for each cohort (e.g., March 1 to March 30).

### Data analyses

The REACH VET program classifies all patients with a REACH VET risk score in the top 0.1% as at high risk. Accordingly for the first objective and independently for each month, we ranked patients by their full model risk score and coded the top 0.1% as the high-risk group. Recall that this risk score is not the standard REACH VET risk score but is produced by re-estimating model coefficients for the combined outcome of death by suicide or suicide attempt. We then created a confusion matrix for the outcome (yes/no) by high-risk status (yes/no) among legal-involved veteran patients and calculated the positive predictive value (PPV) and false negative rate (FNR). Many other metrics for prediction accuracy exist^21^, but we opted for PPV and FNR to be consistent with our results published elsewhere in this issue. Summary values across all five months, including a 95% confidence interval, were obtained through random-effects meta-analysis. Our data use agreements for the mortality data repository and suicidal behavior and overdose reports prevent us from reporting counts lower than 10.

Similar analyses were done for the models trained on the subset of legal-involved veteran patients. Analyses of the full models in our data showed that on average 9.8% of patients in the high-risk groups were legal-involved. To make a fair comparison without theoretically increasing clinician burden or requiring an increase of available clinical resources, we coded the same number of patients – on average 672 – of each legal-involved veteran model as the high-risk group and computed PPV and FNR. To address the first objective of the study we compared summary values for PPV and FNR based on legal-involved veteran models with those for the full models.

To address the second objective of the study we ran similar analyses on legal-involved veteran models that included the additional predictors: family discord, high pain, positive screen on the Columbia Suicide Severity Rating Scale, and prior suicide attempt that resulted in injury. Summary values for PPV and FNR based on these models were compared with summary values for legal-involved veteran models without these predictors.

### Software

Basic descriptive analyses were done in Python 3.11^22^. We used statsmodels version 0.14.2 for Python to train logistic regression models. Random-effects meta-analysis was done with the metafor package for R 4.4.1^23^.

## Results

The mean number of patients included across the five months was 6,863,473 (*SD* 84,416), with an average number of patients who died by suicide or who attempted suicide of 2710 (*SD* 84.1), which equates to 39.5 monthly attempts or deaths per 100,000 or 474 annual attempts or deaths per 100,000. The average number of legal-involved veteran patients across the five months was 42,464 (*SD* 721). Among them, on average 175.8 (*SD* 8.1) patients died by suicide (<10) or attempted suicide, which equates to 415.7 monthly attempts or deaths per 100,000 or 4988 annual attempts or deaths per 100,000.

### REACH VET performance among Legal-Involved Veterans

**Fig. 1:**
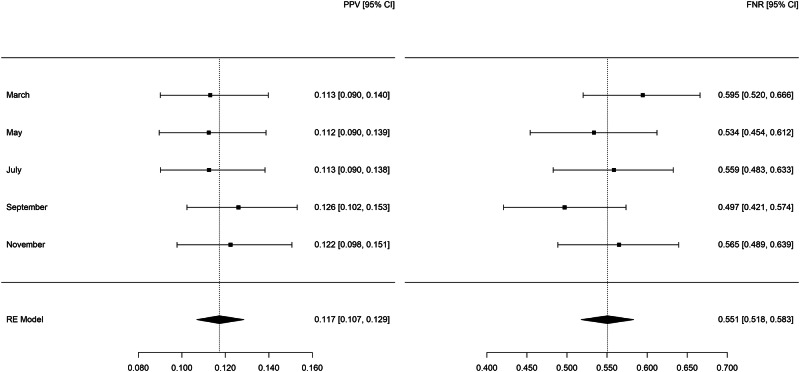
Random-effects meta-analysis of the positive predictive value and false negative rate for legal-involved veteran patients in 2021 as predicted by the full REACH VET model.

Summary statistics for legal-involved veteran patients as predicted by the full models (see Fig. 1) showed a PPV of 0.117 (95% CI: 0.107 to 0.129), indicating that 11.7% of legal-involved veteran patients in the high-risk group died by suicide or attempted suicide. The FNR was 0.551 (95% CI: 0.518 to 0.583), meaning that 55.1% of legal-involved veteran patients who experienced the outcome were in the lower-risk group. Among patients in the high-risk group fewer than 10 died by suicide each month.

**Fig. 2:**
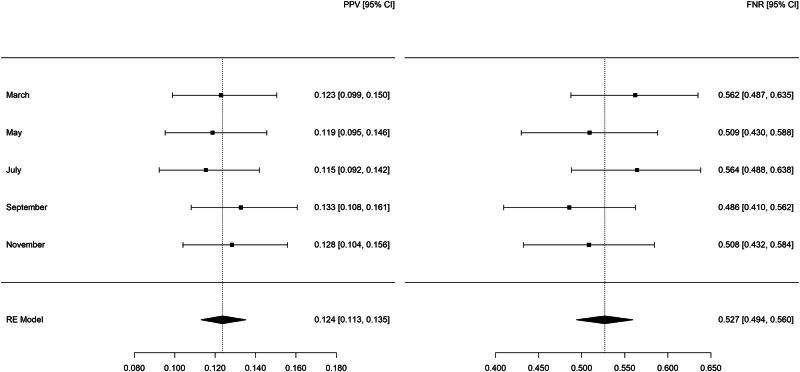
Random-effects meta-analysis of the positive predictive value and false negative rate for legal-involved veteran patients in 2021 as predicted by legal-involved veteran models.

Summary statistics for legal-involved veteran patients as predicted by the legal-involved veteran models (see Fig. 2) showed a PPV of 0.124 (95% CI: 0.113–0.135) and FNR of 0.527 (95% CI: 0.494–0.560). The confidence intervals of the PPV and FNR estimates overlap with the corresponding metrics observed for the legal-involved veteran patients as predicted by the full models, indicating no statistical difference between the two modeling approaches. Among patients in the high-risk group as predicted by the legal-involved veteran models fewer than 10 died by suicide each month.

### REACH VET performance among legal-involved veterans with additional predictors

**Fig. 3:**
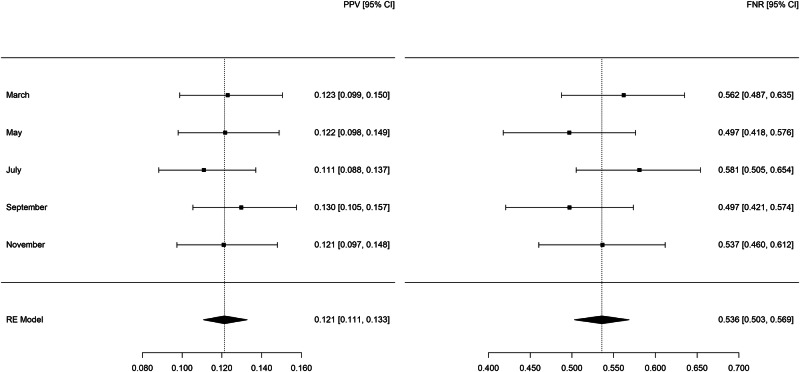
Random-effects meta-analysis of the positive predictive value and false negative rate for legal-involved veteran patients in 2021 as predicted by legal-involved veteran models with additional predictors. Additional predictors included family discord, high pain, positive screen on Columbia Suicide Severity Rating Scale, and prior suicide attempt that resulted in injury.

Summary statistics for legal-involved veteran patients as predicted by legal-involved veteran models with additional predictors (see Fig. 3) showed a PPV of 0.121 (95% CI: 0.111–0.133) and FNR of 0.536 (95% CI: 0.503–0.569). The confidence intervals of the PPV and FNR estimates overlap with the corresponding metrics observed with the legal-involved veteran models without additional predictors, indicating no statistical difference between the legal-involved veteran models with and without additional predictors. Among patients in the high-risk group fewer than 10 died by suicide each month.

## Discussion

The goals of this study were 1) to explore whether retraining the REACH VET suicide risk prediction model for legal-involved veteran patients only would improve its predictive ability for that population, and 2) to explore whether additional predictors associated with being legal-involved would improve predictive ability for that population. For the first goal, we observed modest improved predictive ability as a result of retraining the model in legal-involved veteran patients. Based on summary metrics, the PPV increased from 11.7% to 12.4% and the FNR decreased from 55.1% to 52.7%. These are very small effect sizes that were not statistically significant, likely because the outcome event is still rare, even when combining deaths by suicide and suicide attempts. For the second goal, we did not observe improved predictive ability when adding predictors for family discord, high pain, a positive screen on the Columbia Suicide Severity Rating Scale, and prior suicide attempt resulting in injury to the REACH VET model. In fact, predictive ability was slightly worse in comparison with the REACH VET model without these predictors. These predictors were suggested as potentially important for suicide risk prediction in this population based on an internal quality improvement program. It should be investigated if a different operationalization of the predictors that perhaps aligns more closely with the clinical expertise that informed the quality improvement program could lead to a different outcome.

It is important to point out that these analyses used data generated during a time when the REACH VET program was active. This means that the interventions that are part of the program may have prevented suicide deaths or attempts that might otherwise have occurred in the high-risk group. What little information is known about the effectiveness of the REACH VET program - a 5% reduction in the probability of documentation of a suicide attempt but no effect on deaths by suicide^8^ – suggests that the effect on our current analyses was most likely small. Second, we retrained the REACH VET model for each dataset that we used. This basically provides the best possible performance in terms of accuracy. It should be kept in mind that each of these models when applied to unseen data – that is data the model was not trained on – is likely to have lower performance in terms of PPV and NPV. As our analyses compared models that were trained using the same methods and did not intend to generalize to the best possible model for all data (seen and unseen), we do not expect these limitations to limit our conclusions in any way. Third, our study did not identify all veterans who were legal-involved, as it focused on suicide prevention within the VHA only and used Veterans Justice Programs contact as a proxy for legal involvement. Also, not all legal-involved VHA patients seek support from the Veterans Justice Programs office. More research may be necessary to study the impact of legal involvement on suicide among veterans who do and do not engage with the VHA. Fourth, the additional predictors that we tested were suggested based on clinical expertise. There may be other variables that are associated with being legal-involved that have not been tested in the REACH VET model.

As reported elsewhere in this issue, the ratio of suicide attempts to suicide deaths is more than four times higher for legal-involved patients compared to VHA patients overall. The number of deaths by suicide in our study that were accurately predicted among patients classified as being at high risk for suicide with models that were trained to predict the combined outcome of death by suicide or suicide attempt was very low (<10). While this is disappointing, it is no different for prediction of death by suicide with the original REACH VET model, which has very low sensitivity, as reported elsewhere in this issue. Retraining the REACH VET model on the combined outcome of death by suicide or suicide attempt, is a departure from the original REACH VET model^6^. Beyond producing better predictive performance, most likely because there are more events to train the model on, we would argue that it is also more ethical from a harm reduction point of view. People who attempt suicide experience the stigma of having attempted suicide for the rest of their lives^24^. This makes it more likely that they will at some point die by suicide, as a prior suicide attempt is the most important predictor of death by suicide^9,15^. Many people who attempt suicide require extensive mental health care as well as care for physical health to recover. Moreover, there is a downstream effect on family and friends who themselves become at higher risk of attempts and dying by suicide^25–28^, and the costs in terms of health care and lost productivity are substantial^29–32^.

Our analyses suggest that retraining the suicide risk prediction model that underlies the REACH VET program on the cohort of VHA patients who are involved in the legal system may improve predictive ability of the model for these patients, more so than adding additional predictors associated with being legal-involved. An average increase of 0.7 of a percentage point was observed in positive predictive value as well as an average decrease of almost two percentage points in false negative rate. This is driven by a higher number of suicide attempts that were accurately predicted. We did not see improvement in the number of deaths by suicide that were accurately predicted. While these may seem small gains, with an average of 672 legal-involved veterans patients among the high-risk group as identified by the REACH VET model, these results translate to an additional five patients each month who would receive care under the REACH VET program and contribute to achieving the national goal of zero suicides set forth by the US Department of Health and Human Services^33^. A similar approach for other high-risk patients is worth exploring.

## Supplementary information

Supplementary information

## Data Availability

The United States Department of Veterans Affairs (VA) places legal restrictions on access to veterans’ health care data, which includes both identifying data and sensitive patient information. The analytic data sets used for this study are not permitted to leave the VA network without a Data Use Agreement. For more information, please visit https://www.virec.research.va.gov or contact the VA Information Resource Center (VIReC) at virec@va.gov.
