# Supplementary material for: Retraining the veterans health administration’s REACH VET suicide risk prediction model for patients involved in the legal system
Source: Npj Ment Health Res. 2025 Jul 10;4:29. doi: 10.1038/s44184-025-00143-9 (PMC12246187; doi:10.1038/s44184-025-00143-9)
Supplement: Supplementary file 1 — Supplementary information [file 44184_2025_143_MOESM1_ESM.pdf]

Supplementary Table S1: Model coefficients for the March 2021 REACH VET model with additional predictors, retrained to predict suicide attempt or death for legal-involved VHA patients (n = 43,589) [a].

	b	***SE***	***P***
Intercept	-4.439	1.145	< .001
Age ≥ 80	-6.496	20.43	.75
Male	-0.223	0.291	.44
Married	-0.085	0.220	.70
Race/ethnicity:			
White	-0.381	0.259	.14
Non-White	-0.621	0.300	.04
Region West	0.266	0.175	.13
Service Connected Disability Status:			
>30%	-0.005	0.296	.99
>70%	0.224	0.226	.32
Alprazolam prior 24 months	-0.382	0.637	.55
Antidepressants prior 24 months	0.395	0.269	.14
Antipsychotics prior 12 months	0.207	0.186	.27
Clonazepam:			
prior 12 months	0.074	0.767	.92
prior 24 months	-0.640	0.623	.31
Lorazepam prior 12 months	0.045	0.301	.88
Mirtazepam:			
prior 12 months	0.494	0.440	.26
prior 24 months	-0.554	0.412	.18
Mood stabilizers prior 12 months	0.137	0.188	.47
Opioids prior 12 months	-0.107	0.205	.60
Sedatives or anxiolytics:			
prior 12 months	-0.568	0.341	.10
prior 24 months	0.045	0.283	.87
Statins prior 12 months	-0.215	0.219	.33
Zolpidem prior 24 months	0.602	0.335	.07
Any suicide attempt:			
prior 1 month	0.807	0.294	.006
prior 6 months	1.176	0.349	.001
prior 18 months	1.292	0.304	< .001
Arthritis:			
prior 12 months	-0.040	0.284	.89
prior 24 months	-0.078	0.269	.77
Bipolar I disorder prior 24 months	0.380	0.212	.07
Head or neck cancer:			
prior 12 months	1.460	8.565	.87
prior 24 months	-1.394	8.482	.87
Chronic pain prior 24 months	-0.155	0.191	.42
Depression:			
prior 12 months	0.375	0.332	.26
prior 24 months	0.270	0.377	.47
Diabetes Mellitus prior 12 months	0.250	0.246	.31
Systemic lupus erythematosus prior 24 months	-6.103	19.77	.76
Substance use disorder prior 24 months	0.587	0.265	.03

	b	*SE*	*P*
Any emergency department visits:			
prior 1 month	-0.261	0.427	.54
prior 2 months	0.592	0.272	.03
Any mental health discharges:			
prior 24 months	0.127	0.222	.57
Any mental health treatment:			
prior 12 months	0.489	1.011	.63
prior 24 months	-1.253	1.436	.38
First use during prior 5 years was prior year	-0.211	0.447	.64
Homelessness or services prior 24 months	-0.144	0.185	.44
Interaction terms:			
Other anxiety disorder×personality disorder [b]	0.050	0.215	.82
Male × Marital status divorced	0.026	0.192	.89
Male × Marital status widowed	-0.264	0.751	.73
Suicide attempt with injury prior 12 months:			
yes	-0.174	0.289	.55
unknown	-1.506	0.214	< .001
Positive Columbia Suicide Severity Screen:			
yes	0.754	0.696	.28
unknown	0.027	0.270	.92
High pain score prior 12 months:			
yes	-0.017	0.210	.93
unknown	-0.137	0.264	.60
Family discord prior 1 month	0.196	0.420	.64
Number of days treatment utilization:			
prior 13 months	0.019	0.019	.32
Number of emergency department visits:			
prior 1 month	0.275	0.159	.08
prior 24 months	-0.019	0.014	.17
Number of days outpatient use:			
prior 7 months	0.023	0.022	.30
prior 8 months	0.024	0.023	.31
prior 15 months	0.023	0.019	.23
prior 23 months	-0.008	0.018	.68
prior 1 month squared	-0.001	0.001	.48

[a] The REACH VET model currently used in VHA clinical practice predicts death by suicide only, [b] prior 24 months.
